# Evolutionary Analyses Reveal Diverged Patterns of *SQUAMOSA* Promoter Binding Protein-Like (*SPL*) Gene Family in *Oryza* Genus

**DOI:** 10.3389/fpls.2019.00565

**Published:** 2019-05-08

**Authors:** Hua Zhong, Weilong Kong, Ziyun Gong, Xinyi Fang, Xiaoxiao Deng, Chang Liu, Yangsheng Li

**Affiliations:** State Key Laboratory of Hybrid Rice, Key Laboratory for Research and Utilization of Heterosis in Indica Rice, Ministry of Agriculture, College of Life Sciences, Wuhan University, Wuhan, China

**Keywords:** evolution, *SBP-box* family genes, motif, miR156, *Oryza* genus

## Abstract

The *SPL* (*SQUAMOSA* promoter binding protein-like) gene family is one of the plant-specific transcription factor families and controls a considerable number of biological functions, including floral development, phytohormone signaling, and toxin resistance. However, the evolutionary patterns and driving forces of *SPL* genes in the *Oryza* genus are still not well-characterized. In this study, we investigated a total of 105 *SPL* genes from six AA genome *Oryza* representative species (*O. barthii*, *O. glumipatula, O. nivara, O. rufipogon, O. glaberrima*, and *O. sativa*). Phylogenetic and motif analyses indicated that *SPL* proteins could be divided into two distinct lineages (I and II), and further studies showed lineage II consisted of three clades (IIA, IIB, and IIC). We found that clade I had comparable structural features with clade IIA, whereas genes in clade IIC displayed intrinsic differences, such as lower exon numbers and the presence of miR156 regulation elements. Nineteen orthologous groups of *OsSPL*s in *Oryza* were also identified, and most exons within those genes maintained constant length, whereas length of intron changed relatively. All groups were constrained by stronger purifying selection and diversified continually including alterative gene number, intron length, and miR156 regulation. Subsequently, *cis*-acting element analyses revealed the potential role of *SPL*s in wild rice, which might participate in light-responsive, phytohormone response, and plant growth and development. Our results shed light on that different evolutionary rates and duplication events might result in divergent evolutionary patterns in each lineage of *SPL* genes, providing a guide in exploring diverse function in the rice gene family among six closely related *Oryza* species.

## Introduction

Rice (*Oryza sativa* L.), one of the most important staple foods, provides more than one-fifth of the calories consumed worldwide (Khush, 2003). As an important model organism in monocot plants, *Oryza sativa* belongs to the *Oryza* genus. The genus *Oryza* probably originated within a relatively short time scale about 15 million years ago (MYA), which includes 23 species categorized into 10 genomic types (AA, BB, BBCC, CC, CCDD, EE, FF, GG, HHJJ, and HHKK) (Aggarwal et al., 1997; Ge et al., 1999; Khush, 2003; Vaughan et al., 2003; Jacquemin et al., 2014). As the most important genetic resources for rice breeding, the *Oryza* AA genome contains two cultivated rice (*Oryza sativa* and *Oryza glaberrima*) and six wild species (*Oryza rufipogon*, *Oryza nivara*, *Oryza barthii*, *Oryza longistaminata*, *Oryza meridionalis*, and *Oryza glumaepatula*) (Vaughan et al., 2003). With an abundant infrastructure of high-quality plant genomes, further studies of gene families at the genome scale are now available in the *Oryza* genus especially among those in the AA genome.

Plant DNA binding transcription factors are capable of regulating mRNA transcriptional initiation, which significantly impact a broad range of plant developmental processes and response to environmental changes (Schwechheimer et al., 1998; Henríquez-valencia et al., 2018). Transcription factors have previously been classified into families, such as *MYB* (Baisakh et al., 2016), *bHLH* (Feller et al., 2011), *MADS-box* (Smaczniak et al., 2012), and *SBP-box* families (Klein et al., 1996). It is common knowledge that the *SPL* (*SQUAMOSA* promoter binding protein-like) gene encodes a highly conserved DNA binding domain, which is known as the *SBP* domain (Klein et al., 1996; Cardon et al., 1999). This domain contains two non-interleaved zinc-binding sites consisting of Cys3HisCys2HisCys or Cys6HisCys sequence motif (Yamasaki et al., 2004). As a CCHC-type zinc finger, the first zinc-binding site (Zn1) is essential in the folding of the overall tertiary structure. The second zinc-binding site (Zn2) at the C-terminal site is responsible for the DNA binding (Yamasaki et al., 2006). In addition, the bipartite nuclear localization signal (NLS) motif is highly conserved in the *SPL*s and other families (Dingwall and Laskey, 1991). The first two *SBP*s were discovered in *Antirrhinum majus* (*AmSBP1* and *AmSBP2*) based on their function to interact with the floral meristem identity gene *SQUAMOSA* (Klein et al., 1996).

So far, numerous *SBP-box* genes have been identified and characterized in plants, including *Arabidopsis thaliana* (Cardon et al., 1999), *maize* (Wei et al., 2018), *apple* (Li et al., 2013)*, caster bean* (Zhang and Ling, 2014), and *pepper* (Zhang et al., 2016). *AtSPL14* has been demonstrated to regulate floral transition negatively and display resistance to Fumonisin B1 (Stone et al., 2005). Likewise, overexpression of *AtSPL3/4/5* results in the promotion of the reproductive transition in response to photoperiod and GA signals (Porri et al., 2012; Yu et al., 2012; Preston and Hileman, 2013). Among the 19 identified *OsSPL*s in rice (Yang et al., 2008), multiple essential and divergent developmental processes have been influenced. For example, *OsSPL3* can increase cold stress tolerance (Zhou and Tang, 2018). Chen used near isogenic lines for mapping a minor QTL for heading date, *qHd1*, which contained *OsSPL2* (Chen et al., 2014). Higher expression of *OsSPL16/GW8* promotes cell division and grain filling, which determines grain shape, rice quality, and yield (Wang S. et al., 2012; Wang Y. et al., 2012; Wang et al., 2015). Additionally, *OsSPL14*, which is synonymous with IPA1, reveals a complex network to regulate plant architecture (Jiao et al., 2010; Lu et al., 2013) and promotes both grain yield and disease resistance (Wang et al., 2018). Those findings stressed that *SPL* gene family, of great significance in rice breeding, represents an important strategy to enhance rice yield performance simultaneously.

MircoRNAs are generally a kind of 20–24 nt non-coding small RNAs. They can bind to their complementary mRNAs and reduce protein expression level (Rogers and Chen, 2013). The regulational mechanism of plant miRNA-guided silencing is ancient and significant (Rhoades et al., 2002). Xie identified 11 miR156 targets from rice *SPL* genes and revealed tissue-specific interactions between miR156 and *OsSPL*s (Xie et al., 2006). The overexpression of miR156 delayed moderately in flowering and decreased, obviously, apical dominance through modulating *SPL* genes (Schwab et al., 2005). Nevertheless, it is still unclear whether miR156 regulation retains conserved in the *Oryza* genus and if the *SPL* gene with a miR156 target site expands in rice domestication. Moreover, despite the great progress made in this field, the origin and evolutionary process of the *SBP-box* gene family in *Oryza* have not been largely undefined.

In this study, *SPL* genes from six *Oryza* species, representing the main AA lineage, were identified. In addition, phylogenetic analysis and classification were performed to explore the evolution of the *SBP-box* gene family. Detailed gene information, including the exon–intron structure, the pattern of the conserved motifs, the role of the miR156 target, as well as the divergence of functions were also discussed systematically.

## Materials and Methods

### Identification and Phylogenetic Tree Construction of *SPL* Genes

The protein and cDNA sequences of six representative *Oryza* species, namely, *O. barthii, O. glaberrima, O. glumipatula, O. nivara, O. rufipogon*, and *O. sativa*, were downloaded from Ensembl Plants release 41 (Bolser et al., 2017) and Phytozome v12 (Goodstein et al., 2012). The hidden Markov model (HMM) profile of the *SBP* domain (Accession No. PF03110) was downloaded from the PFAM database (Finn et al., 2014). All these candidate proteins were separately obtained by HMMER 3.2.1 (Finn et al., 2011) and BLASTP (Camacho et al., 2009). Then, the Pfam tool was used to confirm the highly conserved *SBP* domains (Kong et al., 2018). All the DNA sequences and core motifs of the *SPL* genes were mapped to genome data using the GeneDoc software (Nicholas et al., 1997). ProtParam tools in the ExPASy server was used to calculate physicochemical parameters (Gasteiger et al., 2005).

Multiple sequence alignment of full-length *SPL* protein sequences was performed by ClustalW (Thomopson et al., 1994), and an unrooted phylogenetic relationship was constructed by MEGA7 (Kumar et al., 2016) using the neighbor-joining (NJ) method with the Jones–Taylor–Thornton (JTT) model based on 1,000 bootstrap replicates.

### Gene Structure, Motif, and Homology Analysis

Exon/intron site and length data were extracted based on six respective genome annotation GFF files from Ensembl Plants (Bolser et al., 2017). The software MEME Suite 5.0.2 (Bailey et al., 2009) was employed to identify conserved motifs with the maximum number 20. To predict putative functions of identified motifs, the consensus sequences were subjected to search against the Interpro database (Mitchell et al., 2018). The phylogenetic tree combined with motif arrangement was drawn by EvolView v2 (He et al., 2016), and exon/intron structures were shown by TBtools v0.665 proportionally (Chen et al., 2018).

The orthologous groups between diverse *SPL* genes were deduced by OrthMCL (Li et al., 2003). The collinearity relationships were obtained using BLAST search with the default parameters and generated using a procedure in ColinearScan by the MCScanX toolkit (Wang S. et al., 2012). All *Oryza SPL* genes were then classified into various types of duplications with a “duplicate_gene_classifier” procedure.

### Prediction of miR156-Targeted Genes and Substitution Rates Estimation

All mature sequences of miR156 genes were downloaded from miRBase release 22 (Kozomara and Griffiths-Jones, 2014). Binding sites on *SPL* gene transcripts were identified using the online psRNATarget server (Dai and Zhao, 2011) with default settings. The codon sequence alignments of each species *SPL*s were generated using ClustalW (Thomopson et al., 1994). DnaSP v5 software (version 5.10^1^) was used to calculate non-synonymous (Ka) and synonymous (Ks) substitutions and ratios of Ka/Ks (Librado and Rozas, 2009).

### *cis*-Acting Element Analysis and Expression Analyses of *SPL* Genes in Three *Oryza* Plants

The 2-kbp upstream of the transcription start site from each *SPL* coding sequence was examined, which was regarded as putative promoter sequence. Then, the *cis*-elements were analyzed using the PlantCARE program^2^ (Lescot, 2002).

Three transcriptome data (*O. barthii, O. glaberrima*, and *O. rufipogon*) were downloaded from NCBI SRA databases (SRP151515) to investigate the expression profiles of *SPL* genes in six different tissues, roots, tiller base, leaf blades, panicles (<1; 1–5; >5 cm), leaf sheaths, and leaf pulvini. TopHat2 (Kim et al., 2013) was used to map paired reads to their own reference genomes^3^. Then, the gene expression level was calculated by Cufflinks (Trapnell et al., 2012; Kong et al., 2017). The heatmaps were illustrated by TBtools v0.665 (Chen et al., 2018), and the rice anatomogram picture was downloaded from online website Expression Atlas (Petryszak et al., 2016).

## Results

### Identification of *SPL* Genes in Six *Oryza* Genus Species

**FIGURE 1:**
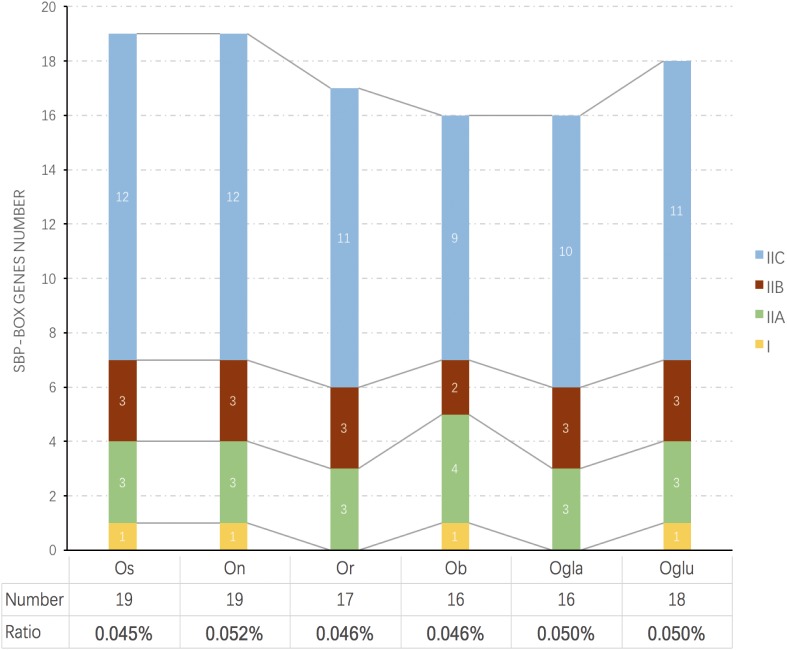
Comparison of the number and ratio of *SPL* genes in six *Oryza* species. The species names with the prefixes “Ob,” “Ogla,” “Oglu,” “On,” “Or,” and “Os” indicate *Oryza barthii, Oryza glaberrima, Oryza glumipatula, Oryza nivara, Oryza rufipogon*, and *Oryza sativa* subsp. *japonica*, respectively. The groups I and II are displayed in different colored boxes. The below chart represents SBP number and their ratios to total protein numbers in different species.

*SBP* transcription factor coding genes in *Oryza barthii* (*Ob*), *Oryza glumipatula* (Oglu), *Oryza nivara* (*On*), *Oryza rufipogon* (*Or*), *Oryza sativa* subsp. japonica (*Os*), and *Oryza glaberrima* (*Ogla*) are convinced by Pfam through searching the *SBP-box* domain. Finally, 16, 16, 18, 19, 17, and 19 *SPL* genes in *Ob, Ogla, Oglu, On, Or*, and *Os* were identified, respectively. Names of putative *Oryza SPL*s were assigned based on chromosomal order in each genome in accordance with previous rice *SPL*s study. These *SPL*s in each species were unevenly distributed on the chromosomes. However, only the *ORGLA08G0230100* gene could not be mapped to any chromosome conclusively and was named as *OglaSPL14* (Supplementary Figure S2 and Supplementary Table S1). The copy number of *SPL*s and the percentage to total genes in each genome were also displayed (Figure 1). We found that *SBP-box* gene family members in Asian cultivated rice (*Os*) showed an obvious gene number expansion compared to wild varieties. Although Asian rice (*Os, Or*, and *On*) had more *SPL* gene copy number than African rice (*Ob* and *Ogla*), the *SPL* number proportion of the latter was larger because of their relatively small genome sizes.

### Comparative Phylogenetic and Motif Composition Analyses

We constructed a NJ phylogenetic tree for the 105 *SPL* genes from six *Oryza* species. In this tree, the *SPL* genes of *Oryza* plants were clustered into two main lineages (I and II), and we observed an apparent difference in the *SPL* gene copy number among these two groups. Only four genes were classified into clade I. Meanwhile, clade II contained the remaining genes, and it was further grouped into three main subclades (IIA, IIB, and IIC), which had several members in each species (Figure 1). Of 101 *SPL* genes in clade II, over 64% gene copies were found in clade IIC, and the number of genes was relatively conserved among the six species: *Os* had 12 genes, and *On, Oglu, Or, Ogla*, and *Ob* had 12, 11, 11, 10, and 9 genes, respectively (Figure 1). In contrast, there were comparatively less than four gene copies in clade IIA. These results indicated that the *SPL* family expansion was mainly the result of duplication of group II genes, especially in clade IIC.

**FIGURE 2:**
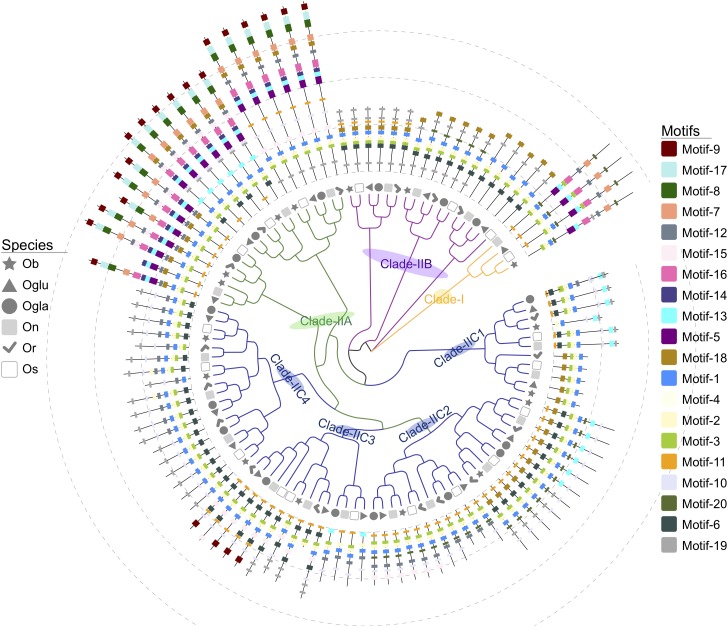
Unrooted phylogenetic tree and motif architectures of *SPL* genes in six *Oryza* plants. The different-colored cluster lines indicate different groups of SPL genes. The different-shaped arcs indicate different species. The motifs, numbers 1–20, are displayed in different colored boxes.

Twenty putative motifs (Figure 2 and Supplementary Table S2) were found through the MEME suite, and these results indicated that the *SBP* domain was composed of three motifs: Zn1 (motifs 2 and 3), Zn2 (motif 4), and NLS (motif 1). The domain structures of *Oryza SPL*s were further analyzed by multiple sequence alignment with full-length protein sequences. As shown in Supplementary Figure S1, the C-terminal zinc finger of both clades I and II had the same C2HC motif, while their N-terminal zinc finger showed different signatures, C4 in clade I and C3H in clade II. Moreover, the *Oryza SPL* proteins in the same clade exhibited similar motif composition. Motifs-5, -7, and -16 were group-specific elements in clade IIA and I, as motif-9 only existed in clade IIA, which made a transmembrane helix. Meanwhile, clade IIC and IIB *SPL* genes contained other unique motifs accept highly conserved *SBP* domains. The Ankyrin repeats (motif-8) were also observed nearby the *SBP* domain in clade IIA, indicating that those domains might mediate protein–protein interactions (Michaely and Bennett, 1992; Li et al., 2006). Most *SPL* genes’ exon sequence in clade IIC possessed a unique motif (motif-10), which could be recognized and regulated by a conserved peptide element ALSLLS of miR156, except for *OsSPL4*, *OsSPL13*, and their orthologous genes (Supplementary Table S2), with binding sites located in the 3′ untranslated regions (UTR) (Xie et al., 2006). We also used the online prediction tool (psRNATarget) to screen miR156-targeting sequences in *SPL* transcripts, and 58 *SPL* genes were indicated as the putative targets. It was comparable with the result of motif-10 prediction that all in clade IIC had conserved functional sites either in the last exon or the 3′-UTR (Supplementary Table S3).

### Homologous Relationship, Gene Structure, and Selective Forces

Homologous genes were mainly classified as orthologous and paralogous types. For one gene family, tandem and segmental duplication events are the main reasons for gene expansion (Coghlan et al., 2005). MCScanX toolkit was used to scan each *Oryza* genome for identifying putative paralogous chromosomal regions. In the *OsSPL* gene family, five homologous gene pairs of *Oryza sativa* (*OsSPL3/12, OsSPL4/11, OsSPL5/10, OsSPL14/17*, and *OsSPL16/18*) were reported to locate within segmental duplication region (Yang et al., 2008). Besides that, five, four, three, four, and four paralogous pairs of *SPL* genes were identified in *Or, On, Ob, Oglu*, and *Ogla*, respectively. Interestingly, we found that paralogs of the *SBP-box* family in Asian rice (*Os, Or*, and *On*) were larger than those of the African rice (*Ob* and *Ogla*) (Supplementary Table S4). Except for one tandem duplicate gene pair (*ObSPL1*/*ObSPL2*), we called the other paralogous genes as ohnologs, which derived specifically from WGD (whole genome duplication).

**FIGURE 3:**
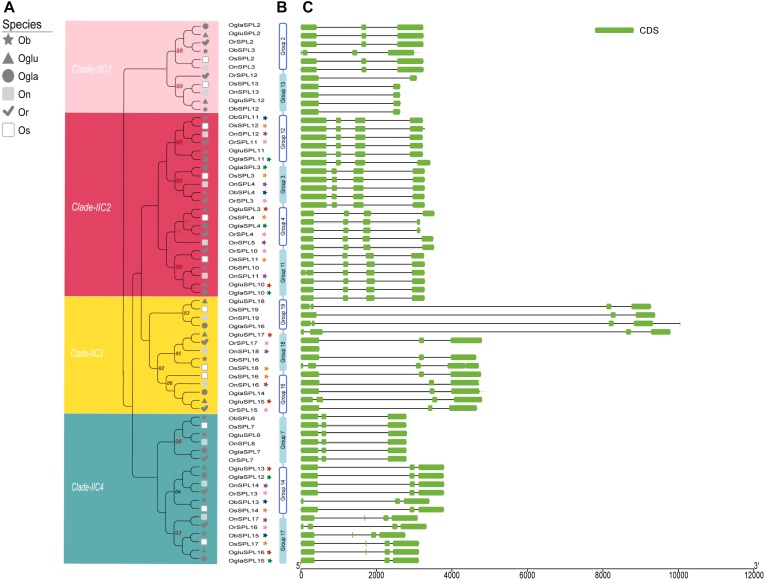
Gene structure of four subgroups and 12 orthologs in clade IIC. **(A)** A phylogenetic tree of IIC was constructed. Details of four clusters are shown in different colors, and the number above the branch indicates the bootstrap values over 50%. Colorful stars display paralogous pairs in each species. **(B)** Orthologous groups in clade IIC. **(C)** Exons–introns and untranslated regions (UTRs) of *SPL* genes. Green boxes indicate exons; black lines indicate introns. The length of protein can be estimated using the scale at the bottom.

In order to understand the evolution pattern of the *SPL* genes in whole *Oryza* AA genus, we used the OrthoMCL software to investigate orthologous target gene pairs with a basic codon substitution model. In total, 19 orthologous groups were identified termed corresponding with *OsSPL* names (Figure 3B and Supplementary Figure S3). Intron/exon numbers might represent splicing variants and were used to classify genes (Roy and Gilbert, 2006). Thus, intron/exon structures of each orthologous group were generated based on genome sequences and corresponding coding sequences (Figure 3A). In clade I and IIA, *SPL*s contained more than 10 exons, while genes in IIB only harbored three exons; according to this, proteins of clade I and IIA had the long C terminus with more than 700 aa residues (Supplementary Figure S3 and Table S1). Despite that exon copy number was constant in clade IIB or clade IIA, the first two introns elongated or shortened in distinct groups. In clade IIC, we found all groups harbored four or fewer exons and divided them into four subclades IIC-1 to -4 based on phylogenetic and ohnologous relationships (Figure 3B). Most genes in one group containing a similar structure (exon/intron number and length) even belonged to different species. There was one exception in group 4, where the last exon of cultivated rice *SPL*s (*OsSPL4* and *OglaSPL4*) possessed a shorter length than other wild rice *SPL*s. In general, introns exhibited significant length change, whereas most exon maintained a relatively constant length during the course of the *Oryza* evolution. Some closely related groups might suffer exon/intron gain or loss event in one clade, such as group 13 lost one exon compared to group 2 (Figure 3C). It should be noted that group 19 had extreme long introns, which included *OsSPL19*. Since *OsSPL19* was suspected of being likely a pseudogene (Xie et al., 2006; Yang et al., 2008), the function of the orthologous group *SPL19* in *Oryza* needs more experimental validation.

To investigate the selective pressure of *SPL*s, we calculated the number of non-synonymous substitutions per non-synonymous site (Ka), synonymous (Ks), and Ka/Ks ratio in each ortholog group. A statistically significant Ka/Ks ratio, equal to 1.0, meant neutral or absence of evolution. Whereas lower than or greater than that represent purifying selection and positive selection, separately. The infinite value of group 18 (Ks = 0) was excluded for computing values; thereby, our findings indicated that mean Ka/Ks values of all groups were lower than 1.0, which meant under purifying selection. Genes in groups 3, 13, and 15 exhibited low Ka/Ks ratio, suggesting significantly strong signs of positive selection. In contrast, a relative weak positive selection sign was found in group 2 (Supplementary Table S5). At the same time, the classification was also performed with evolutionary parameters (Ka, Ks, and Ka/Ks) between two datasets: miR156-targeted genes (clade IIC) and miR156-non-target genes (clades I, IIA, and IIB; might undergo distinct evolutionary rates and selection pressures (Supplementary Figure S4).

### Analyses of *cis*-Elements and Expression Divergence

**FIGURE 4:**
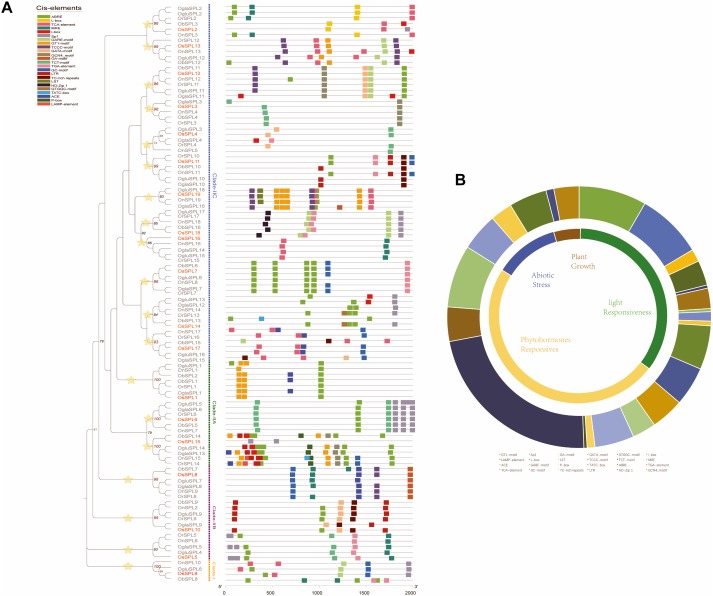
Identification of *cis*-acting elements in all *Oryza SPL* genes. **(A)** *Cis*-acting elements of all *SPL*s based on a phylogenetic tree. The different colored boxes indicate different promoter elements in each *SPL* gene. Yellow stars indicate orthologous groups. **(B)** Pie charts of different sizes represent the ratio of each promoter element in each category.

Regulation of gene expression *via* specific *cis*-elements in the promoter region elements has become a major adaptive mechanism to respond to different environmental conditions (Walther et al., 2007). We searched the PlantCARE database to identify potential *cis*-acting elements in the 2,000 bp upstream promoter regions of *SPL* genes. A large number of *cis*-elements in the promoter regions of *Oryza SPL*s were detected and then were classified into four subdivisions: light responsiveness, plant growth, phytohormone, and abiotic stress response (Figure 4B). More than half of predicted *cis*-elements were classified in the phytohormone response category, including the P-box, TATC-box, GARE motif (gibberellin-responsive elements), and TGA (auxin-responsive element). Among them, ABRE (involved in abscisic acid response) was covered the largest portion (55%), followed by the TCA element (related to salicylic acid; 20%). As for the light responsiveness category, *cis*-acting elements were distributed widely throughout the promoter regions, including a series of elements that participate in part of light responsive (GA motif, GATA motif, LS7, TCT motif, and I-box) MRE (MYB binding site involved in light responsiveness). GT1 motif was the most common (31%), whose proportion was a little higher than Sp1 (30%). In the abiotic stress response category, three main stress-related *cis*-acting elements were identified, known as the GC motif (anoxic-specific inducibility), TC-rich repeats (defense and stress responsiveness), and LTR (response to low temperature). Furthermore, plant growth-related elements HD-Zip 1 (differentiation of the palisade mesophyll cells) and GCN4 motif accounted for 5% (responsible for endosperm expression) in all *cis*-elements, which mainly placed in groups 18, 3, and 12 (Figure 4A).

In our results, *SPL* genes in different orthologous groups were heterogeneous, which might have same functions due to differential types of *cis*-acting elements in their promoter regions (Figure 4A). For example, group 16 only contained two types of *cis*-acting elements involved in the phytohormone responsiveness. Intriguingly, there was a considerable difference between Asian rice (*Os, Or*, and *On*) and the other rice (*Ob, Ogla*, and *Oglu*) in group 11. The former had more three elements (ABRE, GC motif, and TGA element) than the latter (LTR and TC-rich repeats), suggesting more functional regulation of phytohormone and abiotic stress response in the Asian rice (Figure 4A). To explore the expression pattern of wild rice *SPL*s, we also obtained the expression profiles of *SPL* genes in *O. barthii, O. glaberrima*, and *O. rufipogon* from the NCBI SRA databases. In these organisms, a total of six major tissues were examined: roots, tiller base, leaf blades, panicles, leaf sheaths, and leaf pulvini, respectively. We observed that the *SPL*s with miR156-targeted sites represented organ-specific expression patterns, comparing with the *SPL* genes not bound by miRNA (Supplementary Figure S5). Although these transcriptomic data provided us clues to the functional conservation and significance of rice *SPL*s, it remains to be experimentally verified in future studies.

## Discussion

### Evolution of *SPL* Genes in the *Oryza* AA Genus

Previous results reflected that *SPL* genes were specific in the plant and might predate the divergence of the green algae (Guo et al., 2008; Zhang et al., 2015). The ancestor *SPL* gene originally formed two distinct lineages, I and II, by altering the Zn1 from C3H to C4. Clade I contained longer protein length and more exons than clade II. Then, lineage II might be duplicated into three copies, namely, clades IIB, IIA, and IIC, caused by losing exons and introns. Likewise, genes in clade IIA had similar structure properties to clade I with some continuous zero phase introns (Figure 3C), which might be lost more easily. Especially in IIB, the copy number of *SPL*s was relatively small, hinting fewer duplication events occurred. Strikingly, all members in clade IIC had the conserved miRNA target site and even conserved function.

Aimed at dissecting the evolutionary dynamic and function divergence of the *SBP*-*box* gene family in the *Oryza* genus, we identified *SPL* genes in six rice species and determined the phylogenetic relationships of this family. In accordance with the classification of previous research (Guo et al., 2008), we discovered three main clades, IIA, IIB, and IIC, of all 105 *Oryza SPL*s, as outgroup was clade I. Furthermore, 19 orthologous groups of *OsSPL*s were marked in this phylogenetic tree under purifying selection mostly. Group I contained a gene expansion of seven homologs at a smaller scale including six orthologs and one paralogous pair *ObSPL1/ObSPL2*, while the other nine groups had exactly one ortholog in each species. Compared to groups 9 and 19 with barely four species, five groups (groups 3, 4, 5, 13, and 16) had *SPL*s in the absence of one species. For example, *ObSPL*s did not pair with orthologs in other rice sequences in groups 4, 5, and 16. In many high-copy number gene families, such as NB-ARC, F-Box, and B3 gene families, tandem duplication (TD) and gene transposition duplication (GTD) have been reported to frequently occur (Freeling, 2009). In the present study, we identified 25 ohnologous genes, but there was only one paralogous pair (*ObSPL1* and *ObSPL2*) caused by TD considering that the SBP gene family has middle scale of gene copy number (less than 20). As *OsSPL19* has been cognized as a putative pseudogene (Xie et al., 2006), we deduced its orthologs in group 19 (*OgluSPL18, OnSPL19*, and *OglaSPL16*) were pseudogenes. These findings implied that pseudogenization was preserved by loss-of-function mutations in some wild rice, and the acts of *Oryza SPL* genes were redundant and complex. Combined with a detailed comparison of gene structure and motif composition, our results suggested that intra-group genes showed approximate gene structures and conserved motifs in coding regions. While those structural differences among species were less than among the orthologous groups. Thus, we proposed that the *SPL* family is relatively conservative in *Oryza*.

### Functional Diversity in the *Oryza SPL* Gene Family

The *SPL* family has been characterized to have diverse biological processes, governing many fundamental aspects of plant growth and development (Preston and Hileman, 2013). Recent studies have revealed that some group members of the *SPL* gene family regulate various yield-related traits in rice (Wang and Zhang, 2017). For example, *OsSPL8*, known as *OsLG1*, was reported for controlling leaf angle in wild rice and regulating a closed panicle trait in domesticated rice (Ishii et al., 2013). In addition, *OsSPL14/IPA1* promotes panicle branching and grain productivity, which define ideal plant architecture in rice. *OsSPL16/GW8* has been identified as an important QTL improving rice yield and grain quality (Wang S. et al., 2012; Wang et al., 2015). Afterward, *OsSPL13/GLW7* enhances rice grain length and yield by positively regulating cell size in the grain hull (Si et al., 2016). Those researches provided us some hints that *SPL* genes may have great promise for improving many significant agricultural traits in crop species.

In past cases of other species, the *BnaSPL*s (*Brassica napus*) and *ZmSPL*s (maize) in the same phylogenetic clade showed conserved gene structures, which have similar expression profiles with *AtSPL*s (Cheng et al., 2016; Wei et al., 2018). Herein, wild rice *SPL*s had same *cis*-acting elements in the same orthologous group, suggesting that they might perform similar biological tasks to *OsSPL*s. The preliminary *in silico* analysis of gene expression of three wild rice *SPL*s might help us to acquire their tissue-specific expression patterns substantially. Though the wild rice germplasm has been a crucial resource to clone agronomically useful genes for domesticated rice (Doebley et al., 2006), it would be worthy to explore the exact role and possible utilization of wild rice *SPL*s by functional characterization given the lack of related experimental verification on *SPL* genes in wild rice. However, we have to note that redundant orthologous genes do not always predict function considering the negative effect of subfunctionalization (partition original functions) or beneficial effect of neo-functionalization (create new roles) (Force et al., 1999; Teichmann and Babu, 2004). It is widely accepted that the gain of novel function by neo-functionalization is rare, while many redundant genes will become pseudogenes finally at a relatively high probability (Jacquemin et al., 2014). On the other hand, the complexity of *SPL*-related regulatory network also must be taken into account (Teichmann and Babu, 2004; Wang et al., 2015).

## Conclusion

Diversification of *SPL* genes in six *Oryza* genomes was observed from many aspects, including phylogenesis, genomic structure, and the location of the miR156 target site. The *SPL* gene family within the *Oryza* genus displayed size variation between six rice species. All 105 *SPL* genes were divided into lineages I and II, and clade II were further grouped into three clades (IIA, IIB, and IIC) through several rounds of duplication. Genes in one clade experienced similar conserved evolutionary features and *cis*-acting elements, implying similarity of plant biological function potentially. Nineteen orthologous groups of *OsSPL*s in *Oryza* were identified, and all groups suffered relaxed purifying selection and diversified continually including alterative gene number, intron length, and miR156 regulation. Taken together, our results will interpret a comprehensive understanding of the molecular characteristics and evolutionary pattern of the *SPL* gene family in *Oryza* AA genome plants.

## Author Contributions

HZ and WK conceptualized and designed the research. HZ performed most of the analyses and wrote the manuscript. WK carried out the gene expression analysis. ZG, CL, XD, and XF helped in the statistical analyses and revision. YL guided the study.

## Conflict of Interest Statement

The authors declare that the research was conducted in the absence of any commercial or financial relationships that could be construed as a potential conflict of interest.

## Footnotes

1 http://www.ub.edu/dnasp/

2 http://www.dna.affrc.go.jp/PLACE/signalup.html

3 http://plants.ensembl.org/info/website/ftp/index.html

## Supplementary Material

FIGURE S1 Alignment of multiple SBP domain amino acid sequences in *Oryza SPL*s. **(A)** The overall height of each stack represents the degree of conservation at each position, while the height of the letters within each stack indicates the relative frequency of the corresponding amino acid. **(B)** The two conserved zinc-finger structures (C3H/C4, C2HC) and nuclear localization signal (NLS) are indicated on the top. Multiple sequence alignment of full-length protein sequences in the SBP domain was performed.

FIGURE S2 Chromosomal localization of *Oryza SPL*s. **(A–F)** Chromosome mapping of *SPL* genes in the six *Oryza* genomes. Chromosome numbers are indicated at the bottom of each bar. Scale is represented in mega bases (Mb), and lines in the background chromosome indicate all genes within the genomes.

FIGURE S3 Gene structure and othologous groups in clades I, IIA, and IIB. **(A)** Exons–introns and UTRs and orthologous groups in clade **IIA** based on a phylogenetic relationship. **(B)** Exons–introns and orthologous groups in clade **IIB** based on a phylogenetic relationship. **(C)** Exons–introns and orthologous groups in clade **I** based on a phylogenetic tree. Colorful stars display paralogous pairs in each species; yellow boxes indicate exons; and black lines indicate introns. The length of protein can be estimated using the scale at the bottom, and intron phases (0, 1, and 2) are shown.

FIGURE S4 Comparison of the mean Ka/Ks value in miR156-non-target and miR156-targeted genes.

FIGURE S5 Tissue-specific expression analysis of *SPL* genes in **(A)** *Oryza barthii*, **(B)** *Oryza glaberrima*, and **(C)** *Oryza rufipogon*. **(D)** Analyzed tissues included roots, tiller base, leaf blades, panicles, leaf sheaths, and leaf pulvini.

TABLE S1 Summary of information on *SPL* genes in this study.

TABLE S2 Annotation of conserved motifs in *Oryza SPL* proteins.

TABLE S3 The detail information of predicted targets for miRNA.

TABLE S4 Segmentally and tandemly duplicated *Oryza SPL* gene pairs.

TABLE S5 Comparison of the mean Ka/Ks value in each othologous group.
