# Supplementary figures and images for: Evolutionary Analyses Reveal Diverged Patterns of SQUAMOSA Promoter Binding Protein-Like (SPL) Gene Family in Oryza Genus
Source: Front Plant Sci. 2019 May 8;10:565. doi: 10.3389/fpls.2019.00565 (PMC6517846; doi:10.3389/fpls.2019.00565)

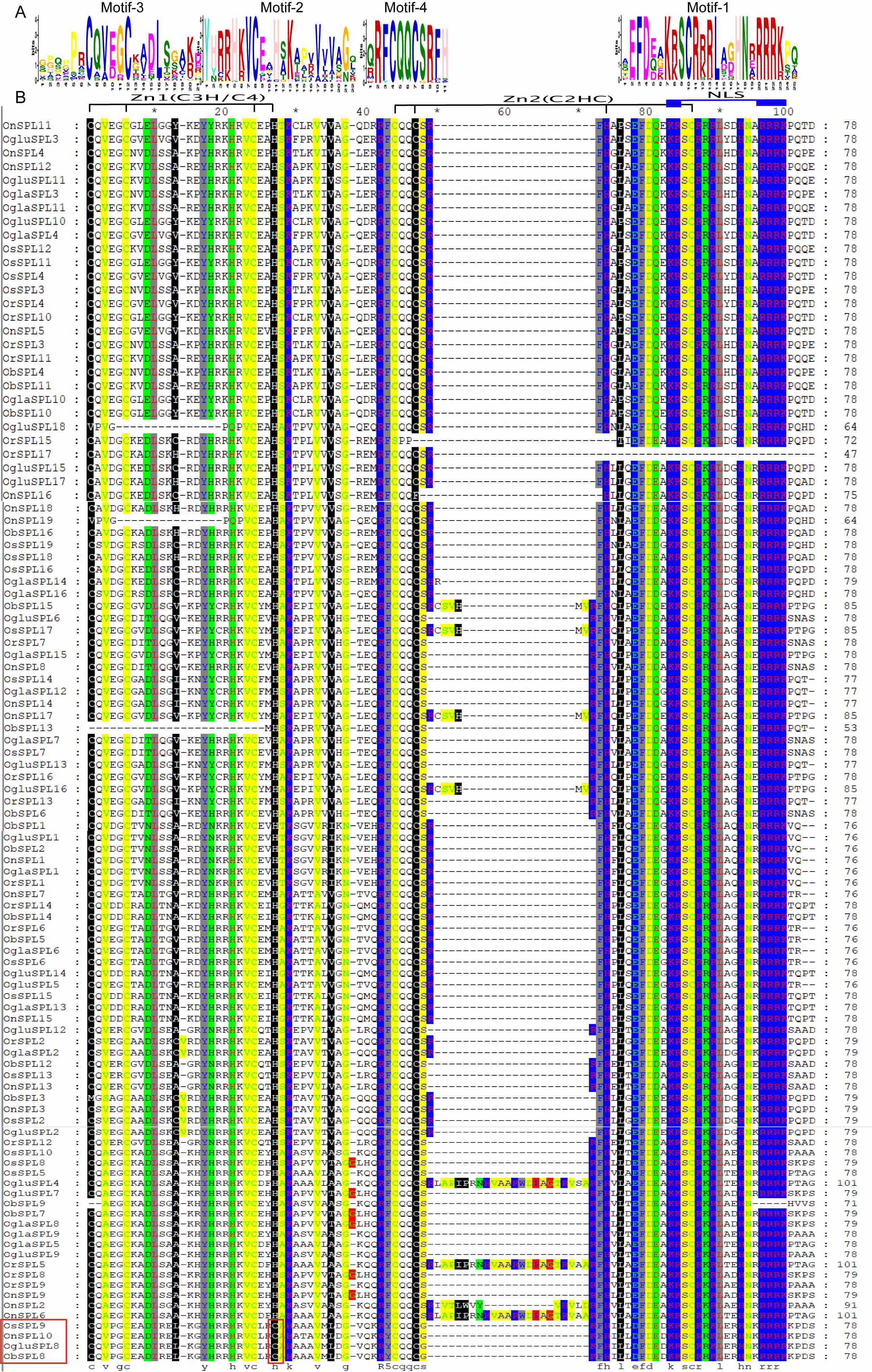

Supplement: FIGURE S1 — Alignment of multiple SBP domain amino acid sequences in Oryza SPLs. (A) The overall height of each stack represents the degree of conservation at each position, while the height of the letters within each stack indicates the relative frequency of the corresponding amino acid. (B) The two conserved zinc-finger structures (C3H/C4, C2HC) and nuclear localization signal (NLS) are indicated on the top. Multiple sequence alignment of full-length protein sequences in the SBP domain was performed. [file Image_1.JPEG]

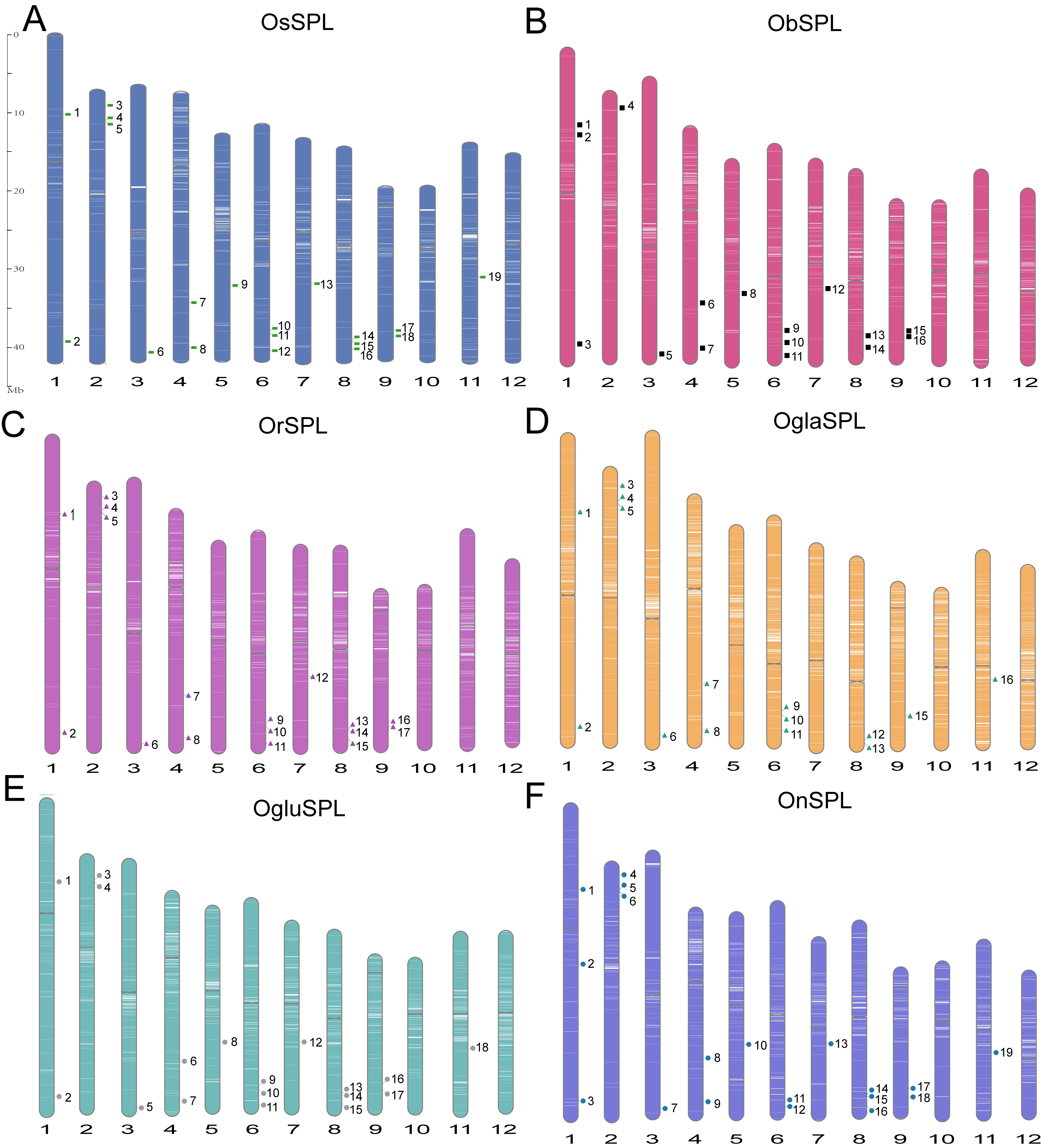

Supplement: FIGURE S2 — Chromosomal localization of Oryza SPLs. (A–F) Chromosome mapping of SPL genes in the six Oryza genomes. Chromosome numbers are indicated at the bottom of each bar. Scale is represented in mega bases (Mb), and lines in the background chromosome indicate all genes within the genomes. [file Image_2.JPEG]

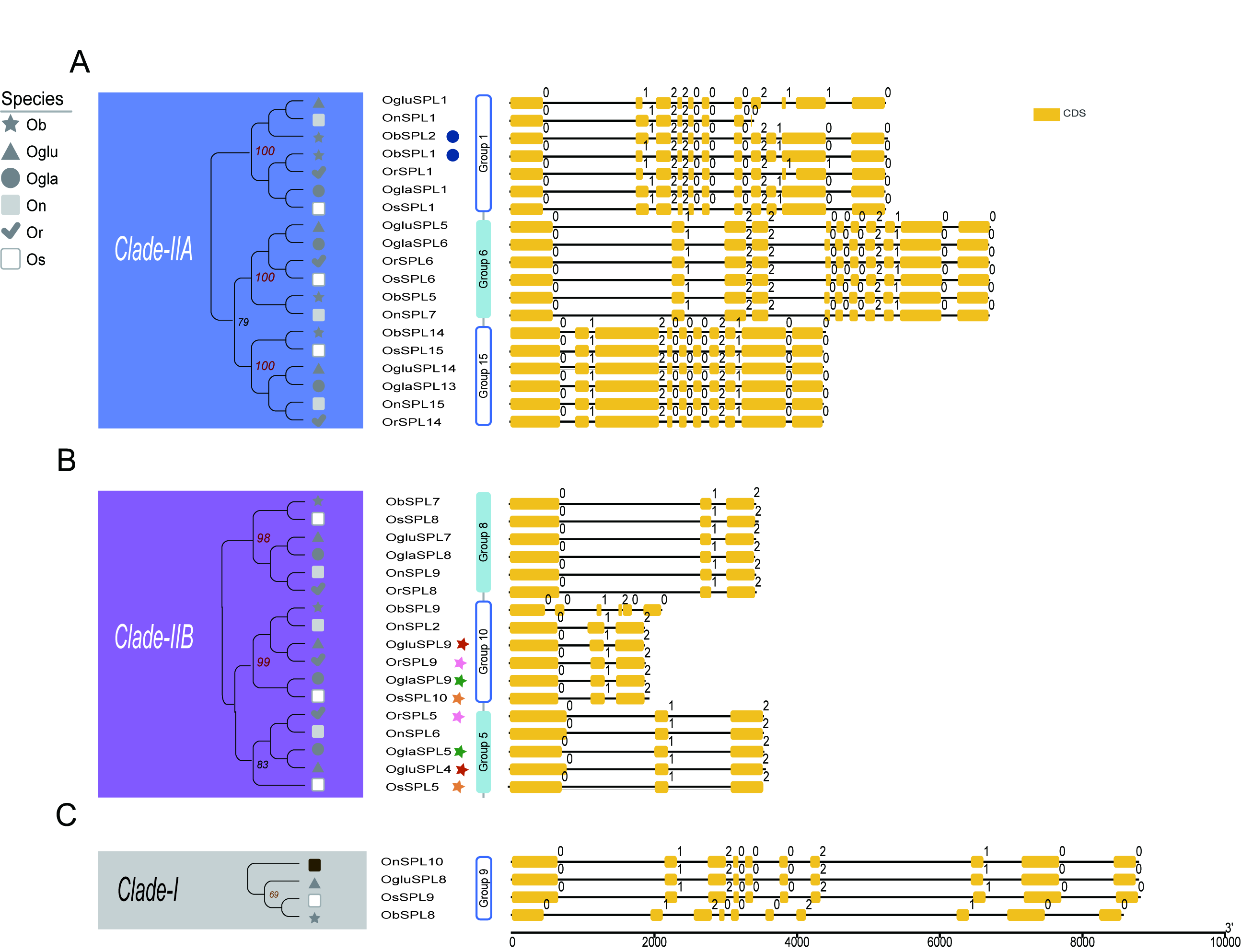

Supplement: FIGURE S3 — Gene structure and othologous groups in clades I, IIA, and IIB. (A) Exons–introns and UTRs and orthologous groups in clade IIA based on a phylogenetic relationship. (B) Exons–introns and orthologous groups in clade IIB based on a phylogenetic relationship. (C) Exons–introns and orthologous groups in clade I based on a phylogenetic tree. Colorful stars display paralogous pairs in each species; yellow boxes indicate exons; and black lines indicate introns. The length of protein can be estimated using the scale at the bottom, and intron phases (0, 1, and 2) are shown. [file Image_3.TIF]

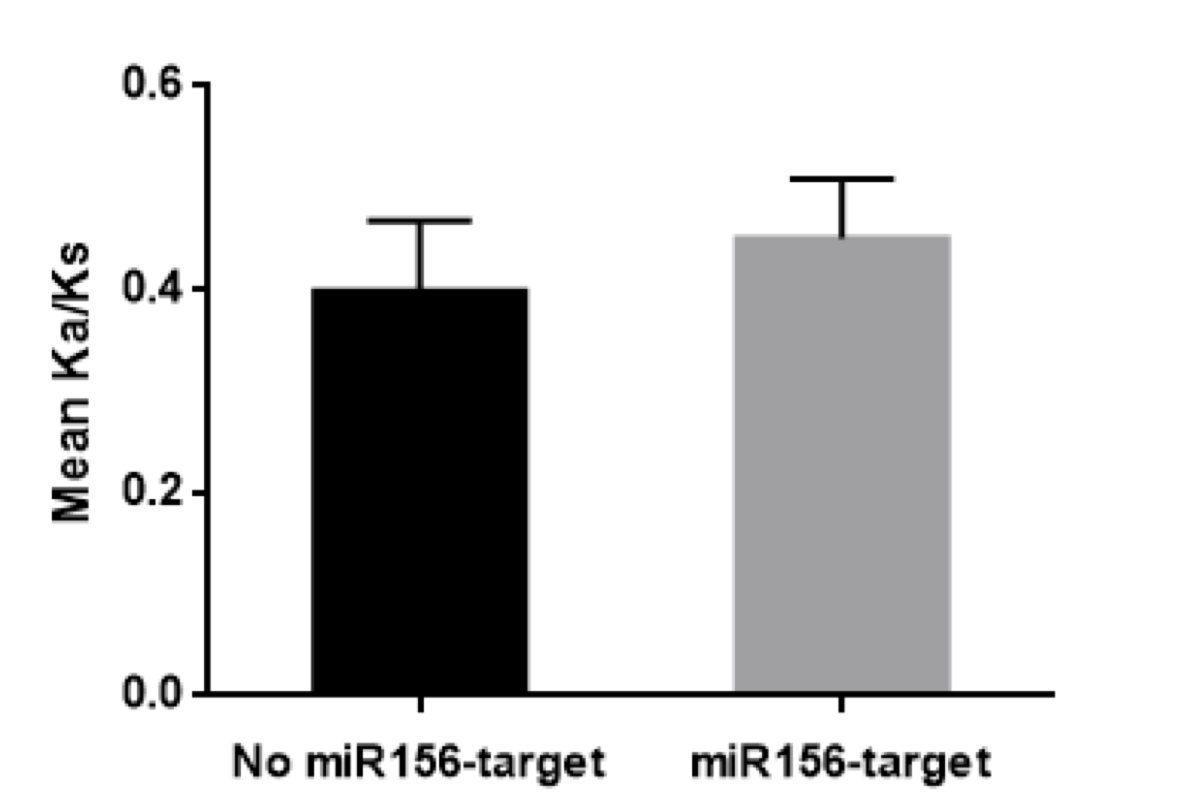

Supplement: FIGURE S4 — Comparison of the mean Ka/Ks value in miR156-non-target and miR156-targeted genes. [file Image_4.TIFF]

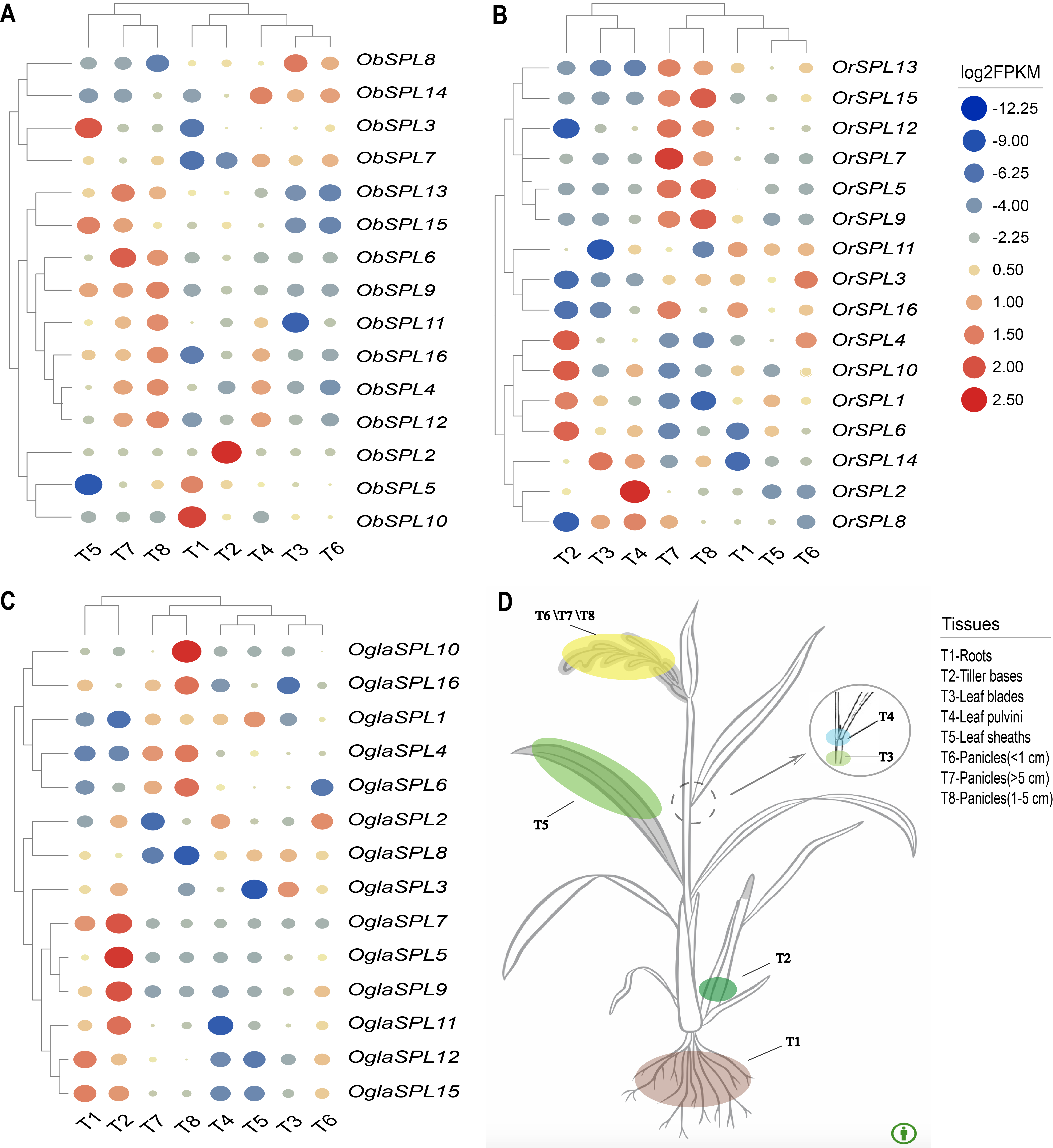

Supplement: FIGURE S5 — Tissue-specific expression analysis of SPL genes in (A) Oryza barthii, (B) Oryza glaberrima, and (C) Oryza rufipogon. (D) Analyzed tissues included roots, tiller base, leaf blades, panicles, leaf sheaths, and leaf pulvini. [file Image_5.JPEG]
